# Using Postmarket Surveillance to Assess Safety-Related Events in a Digital Rehabilitation App (Kaia App): Observational Study

**DOI:** 10.2196/25453

**Published:** 2021-11-09

**Authors:** Deeptee Jain, Kevin Norman, Zachary Werner, Bar Makovoz, Turner Baker, Stephan Huber

**Affiliations:** 1 Department of Orthopaedic Surgery Washington University in St. Louis St. Louis, MO United States; 2 Neoteric Consulting New York, NY United States; 3 Kaia Health GmbH Munich Germany

**Keywords:** lower back pain, digital therapeutics, adverse event, pain, safety, digital health, multidisciplinary pain treatment

## Abstract

**Background:**

Low back pain (LBP) affects nearly 4 out of 5 individuals during their lifetime and is the leading cause of disability globally. Digital therapeutics are emerging as effective treatment options for individuals experiencing LBP. Despite the growth of evidence demonstrating the benefits of these therapeutics in reducing LBP and improving functional outcomes, little data has been systematically collected on their safety profiles.

**Objective:**

This study aims to evaluate the safety profile of a multidisciplinary digital therapeutic for LBP, the Kaia App, by performing a comprehensive assessment of reported adverse events (AEs) by users as captured by a standardized process for postmarket surveillance.

**Methods:**

All users of a multidisciplinary digital app that includes physiotherapy, mindfulness techniques, and education for LBP (Kaia App) from 2018 to 2019 were included. Relevant messages sent by users via the app were collected according to a standard operating procedure regulating postmarket surveillance of the device. These messages were then analyzed to determine if they described an adverse event (AE). Messages describing an AE were then categorized based on the type of AE, its seriousness, and its relatedness to the app, and they were described by numerical counts. User demographics, including age and gender, and data on app use were collected and evaluated to determine if they were risk factors for increased AE reporting.

**Results:**

Of the 138,337 active users of the Kaia App, 125 (0.09%) reported at least one AE. Users reported 0.00014 AEs per active day on the app. The most common nonserious AE reported was increased pain. Other nonserious AEs reported included muscle issues, unpleasant sensations, headache, dizziness, and sleep disturbances. One serious AE, a surgery, was reported. Details of the event and its connection to the intervention were not obtainable, as the user did not provide more information when asked to do so; therefore, it was considered to be possibly related to the intervention. There was no relationship between gender and AE reporting (*P*>.99). Users aged 25 to 34 years had reduced odds (odds ratio [OR] 0.31, 95% CI 0.08-0.95; *P*=.03) of reporting AEs, while users aged 55 to 65 years (OR 2.53, 95% CI 1.36-4.84, *P*=.002) and ≥75 years (OR 4.36, 95% CI 1.07-13.26; *P*=.02) had increased odds. AEs were most frequently reported by users who had 0 to 99 active days on the app, and less frequently reported by users with more active days on the app.

**Conclusions:**

This study on the Kaia App provides the first comprehensive assessment of reported AEs associated with real-world use of digital therapeutics for lower back pain. The overall rate of reported AEs was very low, but significant reporting bias is likely to be present. The AEs reported were generally consistent with those described for in-person therapies for LBP.

## Introduction

Low back pain (LBP) is the leading cause of long-term pain and physical disability in developed countries [1-3]. Nearly 80% of individuals are affected by LBP during their lifetime [4,5]. LBP imposes a major socioeconomic burden on both individuals and industry [6]. In the United States, lost productivity due to LBP, including an estimated 264 million work days lost annually [7,8], contributes to a total economic burden of LBP that exceeds US $100 billion [9,10].

Evidence-based clinical guidelines recommend nonpharmacological approaches, including exercise and mindfulness-based stress reduction care, for individuals experiencing lower back pain [11]. Multidisciplinary pain treatment programs that supplement physiotherapy with mindfulness, exercises, and educational materials are more efficacious at alleviating long-term LBP than physical therapy alone [12-15]. Traditional in-person treatments, however, have a few limitations. They are often costly, which may limit access to those with lower financial means. Furthermore, physiotherapy programs rely on continuous care between appointments and performing exercises independently at home; this reduces adherence, thereby limiting effective treatment [16,17].

Novel interventions, including digital platforms, are becoming increasingly popular to support medical treatment while addressing the limitations of standard in-person treatment options. Digital therapeutics are products that aim to leverage digital, software, or internet-based health technologies to deliver to prevent, manage, or treat medical disorders [18]. Digital therapeutics provide conventional evidence-based interventions on a highly accessible digital platform and in a continuous manner [18]. Digital approaches for LBP are becoming increasingly popular as a means to use the evidence-based, standard of care physical therapy and mindfulness techniques recommended by physicians while increasing accessibility, maintaining program adherence, and reducing costs for users. Multiple digital therapeutic interventions for LBP have been developed, and previous randomized controlled trials (RCTs) have shown that they are effective for reducing pain and disability indices [19-22] and improving adherence to an exercise program [23].

However, few data are available on the safety of these programs. Despite the ease at which digital therapeutics can allow the streamlined collection and recording of safety data from users, some studies fail to report on adverse events (AEs) [19,20,23], while others do not clearly define the methodologies used for reporting [21,22,24]. Additionally, the small sample sizes of the RCTs may have limited the studies from capturing AEs that occur less frequently. AE reporting is critical to identify potential risks associated with the intervention.

The objective of this study was to evaluate the AEs captured with a systematic vigilance process during real-world use of a specific digital therapeutic for LBP, the Kaia App. It was hypothesized that users of the digital therapeutic for LBP would report similar AEs to those in comparable nondigital programs.

## Methods

### Study Design

This study examined the adverse event reporting of all users of a multidisciplinary digital app (the Kaia App) that includes physiotherapy, mindfulness techniques, and education for LBP, from 2018-2019. Relevant messages sent by users via the app were collected according to a standard operating procedure (SOP) regulating postmarket surveillance of the device. These messages were then analyzed to determine if they described an AE. Messages describing an AE were then categorized based on the type of AE, seriousness, and relatedness to the app, and they were described by numerical counts. User demographics, including age and gender, and app use data were collected and evaluated to determine if they were risk factors for increased AE reporting.

### Participants

This retrospective case series included users who were active on the Kaia App from January 2018 to December 2019. Participants had self-reported low back pain. Onboarding criteria for the program have been previously described [25]. The study population in this study consisted of all international users whose interactions were traceable with Kaia’s ticketing system and who were active on the app in 2018 or 2019. Due to data privacy laws, users were given the option to opt in to the use of their personal demographic (age and gender) and app use data, such as active days using the app during the research study. Active days were defined as the number of days in which the users interacted with the app. The rate of AEs per active day of using the app is a metric used to calculate the expected frequency of an AE and to provide a sense of the overall safety profile of the app. This is consistent with the risk management processes for medical devices according to the International Organization for Standardization (ISO 14791). Users were able to withdraw use of the app at any point or to opt out of the collection and storage of personal data.

### Ethical Considerations

The study was conducted with a deidentified data set, which did not contain any electronic personal health information. As such, the study was considered institutional review board–exempt by the Institutional Research Board of the Bavarian Regional Medical Council (2020-1198, Bayerische Landesärztekammer).

### Kaia App Modules

Kaia Health offers a multidisciplinary digital therapeutic solution (Kaia App) for LBP, which has been previously shown to effectively reduce LBP with guided physiotherapy, mindfulness, and educational training [21,26,27]. The Kaia App [25] includes three therapy modules, (1) physiotherapy, (2) mindfulness, and (3) education, with exercises to be performed on a daily basis. The content for each individual user is adapted daily based on the previously completed modules. In this study, users were not obligated to participate in all three modules in a given session. Physiotherapy was limited to up to 5 exercises. The database of 145 exercises was subcategorized into 5 classes based on the targeted body location for that exercise. The exercises recommended were dependent on where the user indicated the most pain. Recommended exercises were adjusted based on ongoing user feedback.

### Reporting of AEs

**Figure 1:**
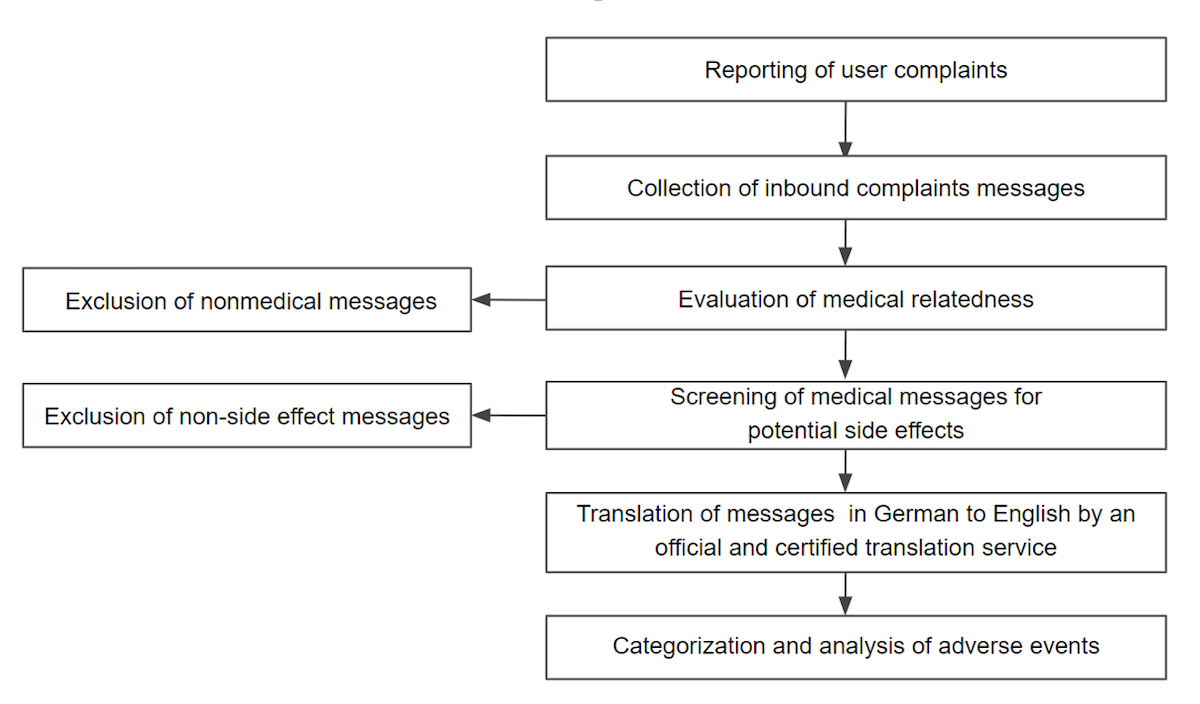
Procedure for the collection and analysis of Kaia App user complaints.

Users regularly corresponded with a personal coach or customer support through the app. Users self-reported AEs to their coach or customer support staff, and the messages were analyzed retrospectively after the users stopped using the app. Users were not specifically prompted to report AEs. All messages indicating potential complaints were tracked in the ticketing system according to an SOP regulating postmarket surveillance of the device (Figure 1). A *complaint* was defined as any written, electronic, or verbal communication that alleged deficiencies related to the identity, quality, durability, reliability, safety, effectiveness, or performance of the app. All messages were screened for medical relatedness and potential side effects by customer support, and if they contained any suggestion of an adverse event, they were forwarded to the Kaia Health medical and quality management team. The process was regulated by the SOP of Kaia Health. The customer support team was trained to label all complaints as either a medical complaint or technical issue. The workflow followed a Kaia internal SOP that includes didactic and supervised learning models. All flagged medical complaints were reviewed by at least one trained, board-certified MD in the field of musculoskeletal pain and a regulatory quality management representative who confirmed each complaint as medically relevant. Any messages written in German were translated to English by a certified translation service, Medax Translation Services (Olching, Germany).

**Figure 2:**
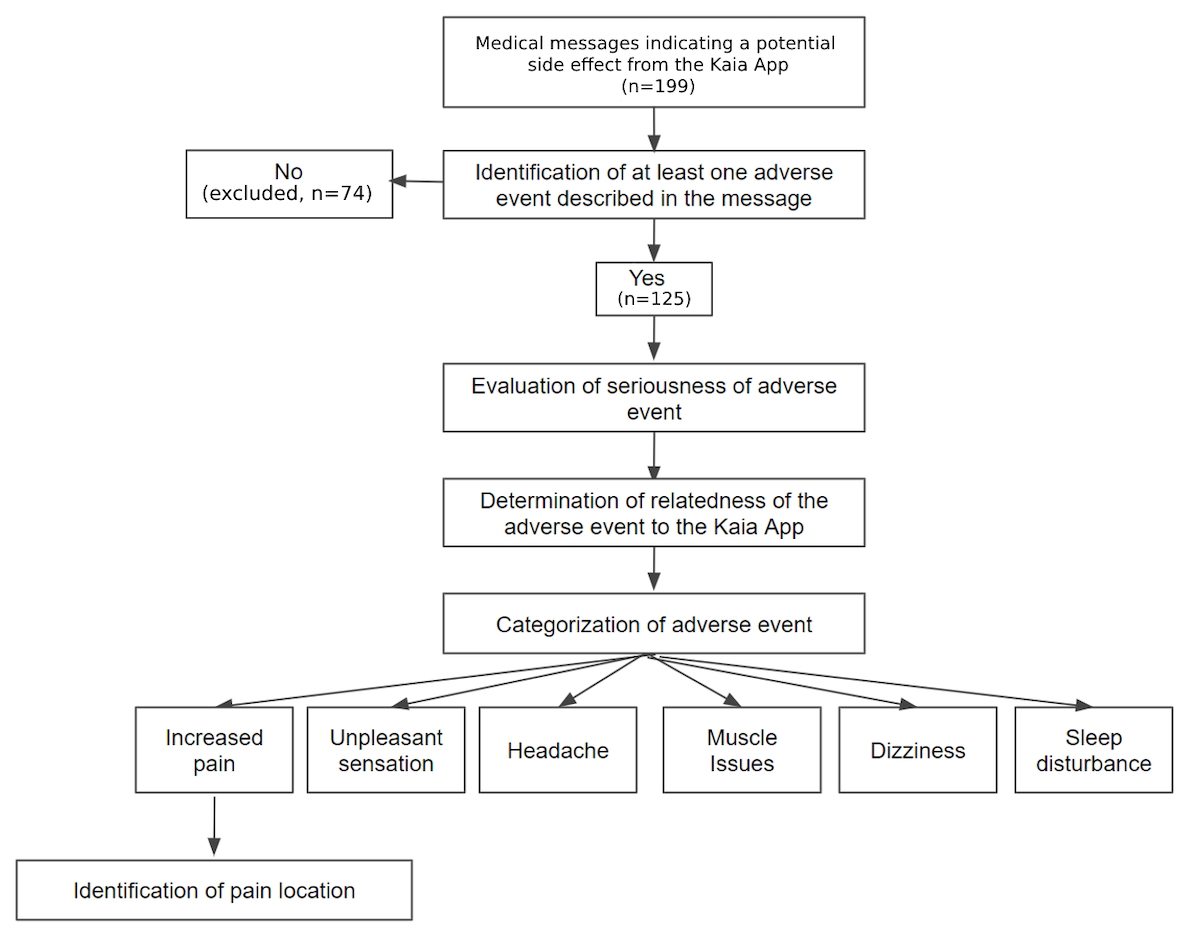
Study process of the medically related message assessment procedure.

A total of 199 medically related messages indicating potential side effects were identified. These messages were then assessed to determine if they described an AE, defined as any untoward medical event. The seriousness, category, and relatedness of the AE to the app was then evaluated (Figure 2), as described below. Two researchers categorized each of the messages independently. Each researcher was blinded to the other’s responses. Discrepancies were decided by a third independent member.

### Adverse Event Seriousness Assessment

The following definitions were used to classify the seriousness of an AE [28]. A serious AE was defined as any untoward medical occurrence that resulted in one of the following outcomes: death, illness/injury requiring hospitalization, events deemed life-threatening, or significant disability. All other AEs were considered nonserious, whether or not they were considered to be related to the intervention. Messages were not considered AEs if they only contained updates on progress, inquiries, or advice on app use.

### Adverse Event Categorization

Previous literature identifying categories of AEs related to exercise and pain management was used to create the classification of AEs [29-31]. The following categories were determined: increased pain, muscle issues, headache, dizziness, unpleasant sensation, and sleep disturbances. Increased pain included any indications of increased pain compared to the user's normal pain level. Muscle issues included muscle-specific discomfort, such as the reporting of muscle cramps, soreness, stiffness, or tightness. Headache included pain in any region of the head. Unpleasant sensation included any reporting of abnormal, uncomfortable sensations, including a feeling of “pins and needles” or unpleasant back cracking noises. Sleep disturbances included disrupted patterns of sleep, including waking up in the middle of the night or difficulty falling asleep.

### AE Relatedness Assessment

To assess the relatedness of reported adverse events, we followed the best practices for AE reporting to the US Food and Drug Administration by registries of postmarket products and applied these principles to the Kaia App digital therapeutic [32,33]. There is no standard nomenclature for describing this relationship, as previous studies have used a variety of terms, such as certainly, definitely, probably, possibly, or likely related or not related [33]. In this study, an AE was considered related to the app intervention if the AE was (1) a known response to similar interventions (ie, biological plausibility) and (2) temporally linked to the intervention. AEs were categorized as definitely or possibly related to app use. AEs were considered definitely related if there was a reasonable, temporal relationship between the AE and the intervention, the AE was consistent with a known or expected response pattern to the intervention, and the AE could not be reasonably explained by the known characteristics of the user’s clinical state. AEs were considered possibly related if the AE followed a reasonable temporal sequence from administration of the study intervention and followed a known or expected response pattern to the intervention, but that could readily have been produced by a number of other factors. If AEs had vague or ambiguous temporal relationships with app use or might reasonably have been a result of a pre-existing condition described in the message, the AE was identified as possibly related.

### Pain Location

The majority of user messages indicating an AE of increased pain specifically identified the location of the pain on the body. AEs indicating increased pain were subcategorized based on location, including pain in the back (including indication of upper and lower back pain and sacroiliac joint pain), neck, shoulder, leg or knee, or other regions, including sciatica, hip pain, or arm pain. If the message did not mention the location of the pain, it was considered nonspecified.

### Statistical Analyses

All statistical tests were performed using RStudio, version 3.5.3 (R Foundation for Statistical Computing). Demographic variables of total app users and users who reported an AE were described by frequency and as distribution (%) within the group. For age variables, odds relative to the age range of 45 to 54 years and 95% confidence intervals were calculated [34]. The relationships between variables (gender, age, and active days) and AEs were analyzed using the Fisher exact test. A 2-sided *P* value <.05 was used to determine statistical significance.

## Results

### Overview of Adverse Event Reporting

A summary of AE reporting from users of the Kaia App for back pain is provided in Table 1. A total of 138,337 users were included. Of the 199 medical-related messages sent by users, 125 reported an AE. These 125 users (0.09% of the total population of 138,337) reported a total of 142 AEs. Among all users in the total population, the app was used for 1,004,430 active days. The rate of AEs was 0.00014 per active day.

**Table 1:** Overview of adverse event reporting on the Kaia App.

Characteristic	Value
Total users on app, N	138,337
Total users reporting an adverse event, n (%)	125 (0.09)
Total adverse events reported, n	142
Total active days using the app, n	1,004,430
Rate of reported adverse events per active day	0.00014

### Demographics

The genders of the all users and the users reporting an AE are displayed in Table 2. Demographic data were available for 74 of the 125 users who reported an AE. No relationship between gender and the reporting of AEs was found (Fisher exact test, *P*>.99).

The ages of all users and users reporting an AE are displayed in Table 3. An odds ratio and 95% confidence interval for AEs was calculated for each age group relative to the age group with the largest number of users (ages 45-54 years). Individuals aged 25-34 years had reduced odds (*P*=.03) of reporting AEs, while those aged 55-65 years (*P*=.002) and ≥75 years (*P*=.02) had increased odds (Fisher exact test).

**Table 2:** Gender demographics of the app users (N=138,337). Demographic data were available for 74 of the 125 users who reported an adverse event.

Gender	Value, n (%)	
	All users	Users reporting an adverse event
Female	76,906 (55.6)	42 (56.8)
Male	57,152 (41.3)	31 (41.9)
Unspecified	4279 (3.1)	1 (1.4)

**Table 3:** Relationship between age and adverse events.

Age (years)	Values				
	All users (N=138,337), n (%)	Users reporting an adverse event (n=74), n (%)	Odds ratio	95% CI	*P* value
<25	9369 (6.8)	1 (1.4)	0.21	0.01-1.35	.15
25-34	25,531 (18.5)	4 (5.4)	0.31	0.08-0.95	.03^a^
35-44	34,826 (25.2)	18 (24.3)	1.20	0.61-2.39	.63
45-54	35,847 (25.9)	15 (20.3)	Reference	Reference	Reference
55-64	22,824 (16.5)	26 (35.1)	2.53	1.36-4.84	.002^a^
65-75	8089 (5.8)	8 (10.8)	1.97	0.74-4.77	.13
>75	1829 (1.3)	2 (2.7)	4.36	1.07-13.26	.02^a^

^a^*P*<.05.

### Categories of Adverse Events Reported and Relationship with App Use

The specific categories of reported AEs are shown in Table 4. Most of the AEs were nonserious, including increased pain, muscle issues, unpleasant sensations, headache, dizziness, and sleep disturbances. All nonserious AEs were determined to be either possibly or definitely related to the digital intervention. One user reported a serious AE, a surgery that occurred during the time period when the individual was using the intervention. Given that we do not have additional information beyond the user messages, we do not know what kind of surgery was performed. This serious AE was rated as possibly related to use of the digital intervention. Users were using the Kaia App as a therapeutic for back pain; therefore, it is uncertain that the injury resulting in surgery was a pre-existing cause of the user’s original back pain or a new symptom.

The anatomical location in which users reported increased pain was then categorized, as shown in Table 5. Due to the self-reporting nature of the AE reporting, many of the users experiencing increased pain did not report the specific location of the increased pain. Of the users who did report a location, back pain was the most common location reported. Users also experienced increased pain in the lower extremities (leg or knee), shoulder, neck, or other body parts including the hip and arms.

Finally, the relationship between the number of active days on the app and the frequency of reported AEs is examined in Table 6. The average number of active days per app user of the total cohort was 7.26 days. AEs were most frequently reported by users who had 0 to 99 active days on the app and less frequently reported by users with more active days on the app.

**Table 4:** Adverse events per category type.

Category of adverse event	Frequency (n=142), n (%)
Increased pain	83 (58.4)
Muscle issues	25 (17.6)
Unpleasant sensation	19 (13.4)
Headache	7 (4.9)
Dizziness	4 (2.8)
Sleep disturbance	3 (2.1)
Surgery	1 (0.7)

**Table 5:** Total adverse events reported per location of increased pain.

Location of increased pain	Frequency (n=83), n (%)
Back	25 (30.1)
Leg or knee	11 (13.2)
Shoulder	11 (13.2)
Neck	8 (9.6)
Other	8 (9.6)
Not specified	27 (32.5)

**Table 6:** Total adverse events reported per active days using the Kaia App. App use data were available for 84 of the 125 users who reported an adverse event.

Active days on Kaia App	Adverse events, n (%)
0-99	51 (60.7)
100-199	18 (21.4)
200-299	6 (7.1)
300-399	6 (7.1)
400-499	2 (2.4)
500-599	1 (1.2)

## Discussion

### Principal Findings

This study provides the first comprehensive assessment of reported AEs associated with real-world use of a digital therapeutic for LBP (the Kaia App). In this retrospective case series, only 0.9% of users reported an AE. AEs were mostly nonserious and included increased pain, muscle issues, dizziness, headaches, and sleep disturbances. The back was the most common location of increased pain reported by users of the app. One serious adverse event, a surgery, was reported; it was determined to be possibly related to the digital intervention, as it could not be determined whether the cause of the surgery was due to the intervention or the underlying condition.

There was no relationship between gender and the reporting of adverse events. Younger users had reduced odds of reporting AEs, while older users had increased odds. On average, users only reported 0.00014 adverse events per active day using the app.

### Comparison With Prior Work

Randomized controlled trials evaluating the use of digital therapeutics for lower back pain have included limited AE reporting [19-24] (Table S1 in Multimedia Appendix 1). The table includes randomized controlled trials that evaluated the use of digital therapeutics for lower back pain and included an analysis of their adverse event reporting. These trials were identified through using comprehensive search terms across the MEDLINE, Embase and Web of Science databases to collect all trials that assessed the use of telehealth interventions available to at-home patients. We found that most of these prior studies did not provide detailed reporting of adverse events; therefore, it is challenging to directly compare the safety of the LBP digital therapeutic in this study to that of other digital-based programs for LBP management.

The AEs reported in this study are comparable to those reported for nondigital forms of the three therapy modules included in the app, including (1) physiotherapy, (2) mindfulness and relaxation exercises, and (3) education for LBP.

All of the AEs in this study were consistent with previously reported AEs related to live exercise therapy. Exercise intervention, while considered safe overall, has been shown to increase the risk of experiencing nonserious AEs in individuals with LBP, but not of serious AEs [35]. Participants who perform either back-focused physical therapy exercises or yoga for LBP [31,36-39] report more AEs than control participants who perform less strenuous nonexercises [40,41]. Most previously reported AEs associated with exercise therapy are musculoskeletal in nature, including increased pain [35] and muscle soreness [42], as well as other nonserious AEs [43] such as headache and dizziness. Previously reported AEs related to yoga include joint pain, increased back pain, sciatica or leg pain, neck pain, abdominal pain, and dizziness [31]. Of note, the rate (0.09%) of reported AEs in this study with a digital app was much lower than what has been reported in prior studies of live exercise therapy for LBP, such as physical therapy (7%-11%) [44] or yoga (7.1%-7.6%) [31,45], although this finding may be limited by the self-reported nature of the AEs collected in this study.

LBP is the second most common reason to visit a primary care physician; it is self-identified, and it is the chief concern upon presentation [46-48]. Thus, the fact that users in this study self-identify as having low back pain makes this study widely generalizable to a broad population.

The pain-related AEs reported in this study have also been reported in prior literature examining mindfulness exercises, but the risk is low. A previous study reported that 10% of individuals with chronic LBP experienced an AE during cognitive behavioral therapy, which was mostly attributed to increased pain from progressive muscle relaxation exercises [49]. However, progressive muscle relaxation techniques have been demonstrated to result in no AEs in individuals with chronic neck pain [50], suggesting that the location of pain before starting the module may influence AE reporting. No study that specifically examined the relationship between breathing exercises and AEs was found.

Finally, the increased number of LBP AEs seen in this study is also consistent with prior literature examining education material related to LBP; however, the risk is low. Literature searches reveal that the existing AE reporting for these interventions is limited, as they are generally considered safe. Individuals given self-care books and newsletters that recommend nonstrenuous stretching routines report very low rates (1.6%) of adverse events, including increased back pain [31]. In another study of participants with LBP assigned to a self-care book treatment, 2.2% participants reported an AE of increased back pain [38].

Next, this study sought to identify risk factors, including age and gender, that may be associated with increased likelihood of reporting an AE. We found that increasing age was a risk factor for reporting an AE. Although moderate to intense exercise has been shown to be safe overall in a healthy population of older people [51-53], older individuals are indeed at increased risk for injury from falls during physical activity [54]. We did not identify a relationship with gender and AE reporting in this study, and to our knowledge, this is the first study to examine gender differences in AE reporting on physical therapy. Although our study focused on demographic risk factors for reporting an AE while using the Kaia App for LBP, future studies should examine additional aspects of back pain that could be risk factors, such as the intensity, duration, and history of LBP [55].

### Limitations

The major limitation of this study is that it was retrospective and relied on self-reporting of possible AEs. Users self-reported AEs to their coach or customer support staff, and the messages were analyzed retrospectively after users stopped using the app. Users were not specifically prompted to report AEs. Overall, this likely resulted in underreporting, and it may explain the low rate of AEs in this study compared to that in prior studies examining live physiotherapy [31]. In particular, the ability to self-report serious AEs is inherently flawed, and more accurate results on the incidence of those events can be more optimally obtained from a prospective study cohort, where planned follow-ups will accurately collect those events. Serious AEs, such as death, cannot be reported by the user, as they would be unable to use the app to report any such event. Although this underreporting would be a serious concern in the tracking of high-risk interventions, by nature, the described intervention is extremely unlikely to cause death or serious AE. Another limitation is that users submitted open-ended messages of variable length and detail to their coach; thus, categorization of AEs was subjective. Although users were not specifically prompted to state the temporal relationship between the AE and app use, many messages did indicate this relationship. To mitigate these issues, two independent researchers classified the medical complaints separately using strict definitions for AE categorization and relatedness, and a third researcher made the final decision on the classification in the event of a discrepancy between the two initial reviewers. To better understand AEs in digital therapeutics, we recommend that a prospective study design be implemented, that users be prompted frequently to report AEs, and that reporting of AEs trigger follow-up questions regarding details.

Finally, due to data privacy laws, users were given the option to opt in to the use of their personal demographic (age and gender) and app use data by the manufacturer; thus, data were missing for some patients who reported adverse events. This may have impacted the analysis of demographics and app use on AE reporting.

### Conclusions

This study serves to emphasize the importance of examining AEs in digital therapeutics for LBP, as these therapeutics are becoming an increasingly popular treatment modality. Future research on digital LBP rehabilitation tools should focus on prospective assessment of AEs using the streamlined nature of data collection in digital interventions to gather safety data from users to identify potential risk factors for negative health consequences.

## Appendix

Multimedia Appendix 1 Review of the adverse event reporting of randomized controlled trials on digital therapeutics for lower back pain management.

## Abbreviations

AE: adverse event
LBP: low back pain
OR: odds ratio
RCT: randomized controlled trial
SOP: standard operating procedure
