# Supplementary material for: Using Postmarket Surveillance to Assess Safety-Related Events in a Digital Rehabilitation App (Kaia App): Observational Study
Source: JMIR Hum Factors. 2021 Nov 9;8(4):e25453. doi: 10.2196/25453 (PMC8663617; doi:10.2196/25453)
Supplement: Multimedia Appendix 1 [file humanfactors_v8i4e25453_app1.docx]

Table S1. Review of the adverse event reporting of randomized controlled trials on digital therapeutics for lower back pain management. Studies were considered to have reported safety if they reported whether or not adverse events occurred during the study.

| **Digital Therapeutic Content** | **Intervention sample size** | **Safety Reporting** | **Definition of Adverse Event** | **Frequency of AE** | |
| --- | --- | --- | --- | --- | --- |
|  |  |  |  | **AE** | **Serious AE** |
| SnapCare App [19] | 45 | No | N/A | N/A | N/A |
| SupportBack (web-based) [23] | 56 | No | N/A | N/A | N/A |
| Hinge App [20] | 113 | No | N/A | N/A | N/A |
| Virtual Reality  (Dodgeball) [24] | 26 | Yes | N/A | 0 | N/A |
| Kaia App [21] | 48 | Yes | N/A | 0 | 1  (unrelated) |
| Wii Fit U exercises [22] | 30 | Yes | undesirable outcome related to the intervention | 0 | 0 |
